# The Role of B Cells in Scleroderma Lung Disease Pathogenesis

**DOI:** 10.3389/fmed.2022.936182

**Published:** 2022-07-04

**Authors:** Stamatis-Nick C. Liossis, Chrysanthi Staveri

**Affiliations:** ^1^Division of Rheumatology, Department of Internal Medicine, Patras University Hospital, Patras, Greece; ^2^Division of Rheumatology, Department of Internal Medicine, University of Patras Medical School, Patras, Greece

**Keywords:** B cell, scleroderma (systemic sclerosis), interstial lung disease, pathogenesis, human

## Abstract

Systemic sclerosis (SSc) is a chronic, autoimmune, multisystem disease characterized by tissue fibrosis that, apart from the skin, may affect the lungs among other organs. B cells have been found in tissue lymphocytic infiltrates; in the lungs are encountered in lymphoid aggregates. The abnormal and hyperreactive B cell in SSc may initiate and perpetuate the fibrotic process via incompletely understood mechanisms. Studies in animal models of SSc have demonstrated that B cell dysregulation is an early event in disease pathogenesis. Functional disturbances of BCR signaling such as decreased inhibitory CD22 signal transduction or augmented CD19-mediated signaling result in prolonged B cell activation. Antagonism of BAFF, a cytokine known for his central role in B cell survival and maturation, not only suppresses the production of fibrogenic cytokines such as IL-6 and IL-10, but also amplifies antifibrogenic cytokine secretion such as IFN-γ and it finally contributes to skin fibrosis attenuation. B cells subsets in SSc patients display several abnormalities. Naïve B cells are increased, in contrast to switched memory B cells that are not only decreased but also activated. Disturbances in the expression of molecules that are involved in B cell tuning have also been described. Interestingly, a distinct B cell population characterized by anergy and exhaustion has been found to be increased in patients with SSc-ILD. Another B cell subset, the CD30^+^GM-Beff, is capable to differentiate monocytes to dendritic cells and is increased in SSc patients with ILD. Of note, patients with SSc-ILD exhibit increased expression of the inhibitory receptor FcγRIIB on naïve and double negative B cells aiming perhaps to counterbalance the abnormal B cell activation. Studies of B cell targeted treatments have demonstrated promising clinical efficacy. Therefore, B cell eliminating therapies could be integrated into the therapeutic armamentarium of patients suffering from SSc-ILD aiming to at least stabilize the fibrotic lung process.

## Introduction

Systemic sclerosis (SSc) is a chronic, autoimmune, multisystem disease characterized by tissue fibrosis affecting the skin, the lungs, and other organs. In patients with SSc, B cells have been found in tissue lymphocytic infiltrates and in the lungs are encountered in lymphoid aggregates (1). Additionally, B cells produce specific SSc-associated autoantibodies (autoAb) and may also present antigens, produce cytokines and regulate T cell function. Based on these, it has been proposed that hyperreactive B cells in SSc may not only initiate but also perpetuate the fibrotic process via incompletely understood mechanisms.

### B Cells May Have a Direct Pathogenic Effect

Recombination activating gene 2 (Rag2) is implicated in the V(D)J recombination in T- and B-cell maturation. Interleukin-2 receptor gamma chain gene (IL2Rγ) is involved in natural killer (NK) cell activity. Therefore, in double knockout Rag2^−/−^IL2Rγ^−/−^ mice the survival of transferred human immune cells was enhanced due to the absence of native B cells, T cells and NK cells, as well as Tregs which may interplay with the human immune response in mice. Peripheral blood mononuclear cells (PBMC) derived from patients with SSc and from patients with granulomatosis with polyangiitis (GPA) were transferred to the immunocompromised Rag^−/−^/IL-2Rγ^−/−^mice (2). Two weeks after the infusion of SSc patient-derived PBMC the recipient mice developed autoAb such as antinuclear antibodies (ANA), as well as cellular tissue infiltrates consisting mainly of B cells in various organs including the lungs. In contrast, passive transfer of PBMC from patients with GPA and from healthy donors did not result in autoAb production or cellular tissue infiltrates. Of note, transfer of PBMC from SSc patients previously treated with RTX failed to induce the pathological findings of human SSc. The study illustrated a potentially direct pathogenic effect of immune cells in SSc in the early stages of the disease progress, assigning a particularly important role to B cells.

### B Cell Implication Is an Early Event in Disease Pathogenesis

A study illustrated that the potential role of B cell may begin during the early stages of the disease course (3). Next-generation RNA sequencing of skin biopsies from patients with early SSc (median disease duration: 1.3 years) yielded high proportions of M2 and M1 macrophages, CD8^+^ T cell, CD4^+^ T cell and B cell signatures (96, 94, 65, 60, and 69%, respectively). Furthermore, analysis illustrated that T and B cell signatures were significantly associated with shorter disease duration even after adjustment for immunosuppression treatment, underscoring thus the potential role of B cells in early disease.

### Cytokine Profile in Th1/Type 1 Effector B Cells Defines the Autoimmune Disease

Type I effector B cells are functionally polarized effectors that produce a Th1-like cytokine profile. A study analyzing 179 patients with 4 different systemic autoimmune diseases including SSc, demonstrated that B cells prosper Th1 differentiation and naïve T cell proliferation resulting in the induction of type I effector B cells that tend to differentiate into plasmablasts (4). This T cell/B cell crosstalk has been associated with the development of a proinflammatory cytokine microenvironment consisting of IL-2, IL-6, CXCL10, and IFN-γ production. This study provides evidence that a shared proinflammatory cytokine profile could define distinct patients of each autoimmune disease. In the proinflammatory group patients with Sjogren's syndrome (SS) and patients with SSc had increased levels of IL-13, patients with SLE had significantly increased levels of IFN-γ and significantly decreased levels of IL-13 and patients with RA had significantly increased levels of IL-8. Scleroderma patients in the proinflammatory group had significantly more often pulmonary, renal and vascular involvement compared to other, “non-inflammatory” patients with SSc (66.5 vs. 13.3%, *p* = 0.0011).

## A) Altered Phenotype of the Scleroderma B Cell

Deranged expression of molecules implicated in B cell regulation is evident in SSc patients. B cells from SSc patients exhibit an activated phenotype (5). Surface expression of the co-stimulatory molecule CD86 was increased in SSc patients, especially in naïve and transitional B cell subpopulations, but no differences were noticed in the expression of MHC II molecules, involved in antigen presentation. The amounts of IL-6 produced by B cells were similar between SSc patients and healthy controls, but IL-10 secretion by B cells was significantly reduced in SSc patients. Most B cells that secrete IL-10 were identified in the transitional B cell subpopulation but the percentage of B cells that secrete IL-10 was reduced in all B cell subsets. Furthermore, a subset of B cells (CD25^high^CD27^high^CD86^high^CD1d^high^) that normally express high levels of IL-10 (as well as TGF-β), was decreased in SSc patients. Disturbances in the expression of regulatory receptors on the surface of B cell subsets of patients with SSc are summarized in Table 1.

**Table 1:** Aberrations in the expression of stimulatory (stim) and inhibitory (inh) receptors on the surface membrane of B cell subsets from patients with SSc.

	**Naïve B cells**	**Memory B cells**	**Transitional B cells**
CD19 stim	↑	↑	↑
CD21 stim	–	–	–
CD40 stim	↑	↑	↑
CD35 inh	↓	↓	↓
CD22 inh	–	–	–
FcγRIIB inh	↑	–	↑
Siglec 10 inh	–	–	–

*↑, Increased; ↓, Decreased; –, no difference between SSc patients and healthy donors*.

### Altered CD19-Dependent Signal Transduction

The tight skin mouse (TSK/+) is a model for human SSc. A tandem duplication in the FBN1 gene in TSK/+ mice is thought to be responsible for the development of skin fibrosis and the production of SSc-specific autoAb. The expression of CD19 on the surface of B cells in the periphery, spleen, bone marrow and peritoneum of TSK/+ mice was similar to wild-type mice (6). However, tyrosine phosphorylation of CD19 was increased by 45% in splenic B cells of TSK/+ compared to wild-type mice. Tyrosine phosphorylation of Vav, a kinase, that mediates downstream CD19 signaling, was also increased in B cells of TSK/+ mice compared to wild-type mice. Lyn, a kinase that is also involved in CD19 tyrosine phosphorylation was amplified by 22% in B cells of TSK/+ mice compared to wild-type mice. Aiming to further examine the role of CD19 signaling in the development of autoimmunity, TSK/+ mice with CD19 deficiency were generated. CD19 loss resulted in reduced activity of Lyn kinase in TSK/+ B cells. This genetic model exhibited an activated CD19-initiated signaling pathway following CD19 ligation and enhanced proliferation of TSK/+ B cells in response to anti-IgM Ab. However, not all signaling pathways of TSK/+ B cells were overreactive because responses to LPS and to anti-CD38 mAb were normal. Therefore, TSK/+ B cells display increased responses specifically via CD19 and the BCR and CD19 deficiency normalizes TSK/+ B cell hyperresponsiveness. Moreover, CD19 deficiency prevented autoAb production and resulted in a decrease of peripheral B cell numbers without significantly affecting B cell development. Phenotypically, CD19 loss was associated with a significantly thinner hypodermal tissue compared to CD19 sufficient mice. Of equal importance, CD19 loss inhibited IL-6 production by TSK/+ B cells. IL-6 induces the production of collagen and glycosaminoglycans by dermal fibroblasts, regulates B cell differentiation and proliferation and it also reinforces autoAb production. IL-4 may also play a role in cutaneous hyperplasia of TSK/+ mice. It has been illustrated that TSK/+ B cells display amplified activation by IL-4 that was reduced by CD19 loss. Collectively, these data suggest that amplified CD19 signal transduction results in chronic activation of B cells leading to autoimmunity and predisposing to fibrosis.

### The Role of CD19 in Lung Fibrosis

A study examined the role of CD19 in the development of lung fibrosis evaluating CD19-deficient mice and human CD19 (hCD19)-overexpressing transgenic mice (7). Bleomycin introduction resulted in consolidation, infiltrates of inflammatory cells, increased collagen deposition and reduced lung function in wild type mice but to a lesser extent in CD19 deficient mice. However, the development of such abnormalities was significantly enhanced in hCD19-transgenic mice. The hydroxyproline content of the lung was decreased in CD19 deficient mice, whereas it was significantly increased in hCD19-transgenic mice.

The B220 isoform of CD45 is a pan B cell marker found in mice. B220 B cell numbers in bronchoalveolar lavage (BAL) fluid were significantly decreased in CD19 deficient mice but were significantly increased in hCD19-transgenic mice illustrating a direct correlation with CD19 expression. Therefore, loss of CD19 prevented B cell accumulation in the lungs following bleomycin treatment. In addition, CD19 deficiency decreased the B220+ B cell count/total cell count, but CD19 overexpression significantly increased the ratio. Of note, the ratio of B220 B cells/total cells in BAL fluid in control (adhesion molecule-deficient) mice was similar to the ratio found in wild-type mice. Therefore, these experiments clearly depict that CD19 expression magnitude was associated with B cell migration into sites of lung inflammation. Additionally, it was shown that migration of B cells into the inflamed lungs was associated with up-regulation of CXCR3, a chemokine induced by CD19 signaling. Bleomycin treatment of mice induced IL-6 secretion; IL-6 was directly correlated with levels of CD19 expression on the surface membrane of B cells. In hCD19 overexpressing mice IL-6 levels were upregulated but in CD19-deficient animals IL-6 levels were decreased. hCD19 overexpressing animals displayed decreased IL-10 levels in contrast to CD19 deficient mice that had increased levels of IL-10. Of note, IFNγ levels were not detectable in CD19 deficient mice, wild-type mice and hCD19-transgenic mice, with or without bleomycin introduction. Finally, it was illustrated that immunoglobulin levels that reflect B cell responses, were strongly correlated with CD19 expression levels after bleomycin treatment. However, although CD19 clearly plays a role in experimental SSc models, a trial of inebilizumab, a mAb targeting CD19 did not seem to improve lung function in patients with SSc-ILD (8).

### Disrupted CD22 Regulation Affects CD19 Activation Status

CD19 overexpression was associated with IgG anti-topo I autoAb production; even higher levels of expression of endogenous CD19 resulted in even higher IgG and IgM anti-topo I autoAb levels (9). Human CD19-trnsgenic (TG)-1 mice and CD19-TG-4 mice were previously backcrossed more than 4 generations onto the TSK/+ mice. Breedings of CD19TG+/– TSK/+ mice resulted in CD19TG1 and CD19TG-4 TSK/+ mice. IgG anti-topo I production was x7.9-fold in CD19TG-4+/–TSK/+ mice and x20-fold in CD19TG-1+/+TSK compared to TSK/+ mice. Similarly, IgM anti-topo I production was x2.7-fold in CD19TG-4+/–/TSK+ mice and x12-fold in CD19TG-1+/+/TSK+ compared to TSK/+. Therefore, overexpression of CD19 in 2 different TSK genetic variants regulates anti-topo I autoAb production levels. Regarding skin fibrosis, it was depicted that CD19 deficiency may attenuate skin fibrosis, but, contrastingly, CD19 overexpression did not amplify skin fibrosis. Evidence raised the possibility of intristic B cell signaling abnormalities in TSK/+ mice. Circulating B cells from TSK/+ mice express lower surface IgM levels, but higher MHC class II levels compared to wild type B cells. Moreover, TSK/+ mice display a hyperreactive B cell phenotype. It has been demonstrated that TSK/+ B cells exhibit a rapid and sustained increase of the intracellular calcium concentration [Ca^2+^] following BCR ligation compared to wild-type B cells. To explain this augmented BCR-initiated signaling, levels and activities of signal transducing tyrosine kinases and phosphatases were sought, but no abnormalities were reported. Nevertheless, CD22 phosphorylation was reportedly decreased in TSK/+ B cells, while BCR ligation resulted in a modest only, CD22 phosphorylation, underscoring that there is a dysregulated CD19/CD22 loop in Tsk/+ B cells. Therefore, disturbances of B cell signaling events, and particularly of those related to CD22 pathway may participate in the development of autoimmunity in TSK/+ mice.

### CD22 and CD72

CD22 and CD72 are B cell-surface molecules that inhibit BCR-mediated signaling. Deficiency of both CD22 plus CD72 in murine knockout models significantly decreased dermal thickness compared to wild type mice (*p* <0.05) (10). Additionally, α-ASMA (+) myofibroblasts were significantly decreased in CD22 and CD72 deficient mice compared to wild type mice. Thus, deficiency of CD22 or CD72 or both attenuated skin fibrosis induced by bleomycin administration.

Lung fibrosis in mutant and wild type mice was also examined. Lung fibrosis scores were significantly better in mutant mice (CD22-deficient, CD72-deficient and CD22 and CD72 double-deficient mice) compared to wild type mice (mice without CD22 or CD72 deficiency). Thus, deficiency of CD22 or CD72 or both decreased bleomycin-induced lung fibrosis. CD22^−/−^, CD72^−/−^ and CD22^−/−^CD72^−/−^ mice transfused with B cells from wild-type mice demonstrated skin and lung fibrosis comparable to that encountered in wild type mice. Moreover, leukocytes, including T cells and macrophages, profibrotic cytokines such as IL-6, TNF-α, IL-1β, IL-13, TGF-β, and CTGF and chemokines such as ICAM-1 and CXCL2 were all reduced in mutant nice compared to wild type mice. Furthermore, an isolated deficiency of CD22 or of CD72 was sufficient to attenuate lung and skin fibrosis, because CD72 blockade in CD22^−/−^ animals did not offer any additional benefit.

### Role of FcγRIIB

A study examined the role of FcγRIIB, a B cell surface receptor for the Fc region of IgG that down-regulates BCR signaling, in the development of a murine bleomycin-induced model of SSc (11). B220+B cells were significantly increased in the skin of FcγRIIB deficient mice compared to the skin of wild type mice. Additionally, FcγRIIB deficient mice demonstrated higher levels of TNF-α and IL-1β production and increased expression of ICAM-1, CCL3, and CXCL2 in the skin compared to wild type animals. Furthermore, an adoptive transfer of splenic CD19^+^B cells and CD19^−^ non-B cells from FcγRIIB deficient mice to wild type mice resulted in lung and skin fibrosis attenuation. Therefore, experimental data suggest that FcγRIIB may play a role in the SSc-related fibrotic process.

### Increased Expression of FcγRIIB on the Surface of Specific B Cell Subsets

Increased CD27^−^IgD^+^ naïve, reduced CD27^+^IgD^+^ pre-switched memory and increased CD27^−^IgD^−^ memory B cells were found in SSc patients compared to controls. Flow cytometry of blood samples from 76 SSc patients and 59 healthy controls demonstrated an increased expression of the inhibitory receptor FcγRIIB levels on naïve (CD19^+^IgD^+^CD27^+^) and double-negative (DN) CD19^+^IgD^−^CD27^−^ memory B cells in SSc patients compared to controls (12). FcγRIIB expression on naïve B cells was increased in patients with ILD treated with glucocorticoids and in patients with a history of intravenous cyclophosphamide (IV CYC) treatment. FcγRIIB expression on switched memory B cells was increased in patients with ILD even without any treatment. There was no significant correlation of FcγRIIB expression levels with skin thickness. Therefore, FcγRIIB expression on naïve and DN memory B cells was increased in SSc patients with ILD specifically. TLC, FVC and DLCO were decreased in patients with high FcγRIIB expression (*p* < 0.005, *p* < 0.01, *p* < 0.05). Apart from ILD, cardiac involvement was similarly over-represented in patients with high FcγRIIB expression on DN memory B cells.

The levels of FcγRIIB expression on DN memory B cells may correlate with disease severity, since the European Scleroderma Group-Activity Index (EScG-AI) was significantly increased in patients with higher levels of FcγRIIB compared to those with normal FcγRIIB levels on the surface of DN memory B cells. FcγRIIB expression levels on pre-switched memory, DN memory and switched memory B cell counts after IV CYC treatment decreased compared to pre-treatment levels, in parallel with ILD improvement. Thus, FcγRIIB expression levels on the surface of certain B cell subsets might serve as a marker of ILD and of disease activity.

## B) Altered B Cell Subsets in SSc

B cells represent a quite heterogeneous population that includes diverse subsets characterized by their membrane phenotype and/or their cytokine secretion profile. A study of 31 patients with SSc and 53 healthy controls demonstrated altered frequencies of B cell subsets in the peripheral blood of SSc patients. More specifically, there was an increased percentage of CD19^+^ B cells in SSc patients, but the percentage of transitional B cells was increased compared to healthy controls (5). Another study showed that in patients with SSc, marginal zone, memory and switched memory B cells were reduced compared to healthy donors (13). In contrast, mature naïve, naïve and CD21^lo^CD38^lo^B cells were significantly expanded compared to healthy donors. CD21^lo^CD38^lo^B cells are characterized by a very low expression of CD21 and low levels of CD38. There was an increased expression of activation markers, such as CD80, CD95, and HLA-DR on the surface membrane of B cells from some B cell subpopulations. More specifically: (i) increased expression of CD80 was reported on marginal zone, mature naïve and switched memory B cells, (ii) increased expression of CD95 on marginal zone, mature naïve and the total B cell population and finally (iii) increased HLA DR expression on the surface of marginal zone B cells. Of note, expression of the apoptosis regulator Bcl-2 was decreased in B cells of SSc patients, particularly in marginal zone B cells. Patients with SSc-ILD have decreased numbers of B cells that secrete IL-10 (B10 cells) when stimulated via a short activation protocol (with the oligodeoxynucleotide CpG for 5 h), but not when activated via a long stimulation protocol (with CpG and CD40L for 48 h), suggesting thereby that B10 cells and not their precursors may be also implicated in the ILD pathogenesis.

### Increased Activated Switched Memory B Cells

Memory B cells depending on their IgD expression levels are further divided into switched (CD19^+^CD27^+^IgD^−^) and non-switched (CD19^+^CD27^+^IgD^+^) memory B cell subpopulations. The percentages of unswitched (CD19^+^IgD^lo^CD27^+^CD38^+^), resting switched (CD19^+^IgD^−^CD27^+^CD38^+^CD95^−^) and activated switched memory (CD19^+^IgD^−^CD27^+^CD38^−^CD95^+^) B cells have been reported to be significantly decreased in SSc patients compared to healthy donors (14). The percentage of activated switched memory B cells was found to be increased in dcSSc patients, in anti-topo I (+) patients and in those with lung involvement (*p* = 0.003) suggesting a correlation of this subset with the severity of SSc disease.

### Reduced Non-switched Memory B Cells

Naïve B cell counts were increased in SSc patients compared to controls (15). However, the proportion of memory B cells was reduced and more specifically that of non-switched memory B cells was reduced. Patients with diffuse SSc exhibited higher levels of DN memory that lack IgD and CD27 and switched memory B cells compared to patients with limited SSc (*p* = 0.031 and *p* = 0.025, respectively). Additionally, CD95^+^ DN memory and CD95^+^CD27^+^ memory B cells were increased in patients with diffuse SSc compared to patients with limited SSc (*p* = 0.045 and *p* = 0.038, respectively). Perhaps the imbalance in B cell repertoire is involved in SSc pathogenesis.

### Defective B Cell Tolerance Checkpoints

Newly emigrant/transitional B cells (CD19^+^CD21^lo^CD10^+^IgM^high^CD27^−^) producing polyreactive antibodies were significantly expanded in SSc patients suggesting a defect in central B cell tolerance (16). Mature naïve B cells (CD19^+^CD21^+^CD10^−^IgM^+^CD27^−^) producing Hep-2-reactive antibodies were also increased in SSc patients compared to healthy donors suggesting an additional defective peripheral B cell tolerance checkpoint. Therefore, incomplete removal of autoreactive B cells may result in the production of self-antigen specific B cells that may implicated in the tissue fibrotic process in SSc.

### Defective Regulation of Transitional IL-6^+^ B Cells

SSc patients have more transitional (CD24^high^CD38^high^) B cells compared to healthy subjects (17). Perhaps more importantly, patients with severe lung disease had more peripheral IL-6^+^ B cells compared to patients with mild disease, and patients with diffuse SSc had more IL-6^+^ B cells compared to patients with limited SSc. However, the differences of previous comparisons were not statistically significant. IL-6 producing transitional B cells may reflect a defect of the checkpoint mechanisms that regulate autoreactive T1-T2 maturation. Stimulation of PBMC enriched in B cells via TLR-9 alone or via TLR-9 along with CD40 resulted in the production of less IL-10 compared to healthy donors. Additionally, transitional B cells were resistant to apoptosis following BCR signaling. Levels of IgM anti-Scl-70 positive transitional B cells were increased in patients with anti-Scl-70 autoantibodies compared to those they did not have anti-Scl-70 autoantibodies. It seems that abnormal regulation of transitional IL-6^+^ B cells in patients with SSc may contribute in the disease pathogenesis.

## C) Distinct B Cell Subsets Associated with SSc-ILD

### Decreased B Regulatory Cells

The frequency of IL-10 producing Breg cells was reduced in the peripheral blood of patients with SSc compared to healthy controls (18). The absolute number of Breg cells was significantly lower in SSc patients compared to disease-control and healthy individuals. For example, frequency of Breg cells in dermatomyositis patients was similar to that encountered in healthy controls. Furthermore, frequencies and absolute numbers of CD24^hi^CD27^+^ that are characterized by a high expression of CD24 and a moderate expression of CD27 were decreased in SSc patients compared to controls.

SSc patients with decreased Breg cells had more frequently ILD. It has been proposed that decreased Breg cells might accelerate autoimmunity in SSc. Breg cell percentages were correlated negatively with the titer of anti-topo I and anti-centromere autoAb in SSc patients. Treatment significantly increased circulating Breg and CD24^hi^CD27^+^ B cells in patients with dcSSc compared to pre-treatment levels. It has been also reported that Breg cells of patients receiving immunosuppressive therapy were numerically, but not significantly increased compared to patients that did not. Thus, Breg cell levels may participate in the development of SSc.

### Granulocyte-Macrophage Colony-Stimulating Factor (GM-CSF)-Producing Effector B Cells (GM-Beffs)

GM-CSF is a proinflammatory cytokine involved in the differentiation of monocytes to myofibroblasts and macrophages and promotes fibrosis in SSc. GM-CSF production is mainly a property of CD19^+^CD27^+^ memory B cells. GM-CSF as well as IL-6 mRNA were up-regulated when B cells were stimulated with IL-4 (a Th2 cytokine), but not upon B cell stimulation with IFN-γ or IL-17 (19). IL4R was induced on the surface of stimulated B cells and consequently IL-4 might have autocrine effects on B cells. Tofacitinib introduction suppressed the expression of GM-CSF mRNA and protein in memory B cells induced by IL-4, but not by TGF-β. The number of GM-Beffs was clearly increased in patients with SSc compared to healthy controls. The number of GM-Beffs in diffuse SSc, limited SSc patients, with and without concomitant ILD were 164.2 ± 257.0, 54.0 ± 47.0, 150.6 ± 245.4, and 55.8 ± 44.3 cells/100 μl, respectively, *p* < 0.05. Thus, GM Beffs may be involved synergistically with IL-4 in SSc pathogenesis via the induction of dendritic cells.

### TIM-1 Defines a Specific B Cell Subset in SSc-ILD

TIM-1 (T cell Ig and mucin domain protein 1), a Breg marker was reportedly increased on transitional (CD19^+^ CD24^hi^CD38^hi^) compared to naïve (CD19^+^ CD24^med^CD38^med^) and memory (CD19^+^ CD24^hi^CD38^med^) B cells in patients with SSc (20). TIM-1 was expressed in all B cell subsets except plasmablasts (CD19^+^ CD24^−^CD38^hi^). Reduced frequency of B cells expressing TIM-1 was only reported in transitional B cells in SSc patients. Investigators found a direct correlation between TIM-1 expression and CD19 on the surface of transitional B cells, as well as a significant increase in TIM-1 expression upon activation of BCR and TLR9 in all B cell subsets. Importantly, TIM-1 expression levels on transitional B cells were higher in patients with dcSSc and worse lung function, expressed as a lower DLCO. TIM-1^+^ B cells from healthy subjects strongly suppressed the expression of proinflammatory cytokines from co-cultured CD4+ T cells such as IFN-γ, TNF-α, and IL-17 compared to TIM-1^−^ B cells. On the contrary, TIM-1^+^ and TIM-1^−^ B cells did not intensively suppress CD4+ T cells like they did in healthy individuals. It was also proposed that TIM-1 is associated with a subpopulation of Bregs different from transitional B cells. Thus, abnormalities in TIM-1^+^ B cells may play a role in SSc pathogenesis.

### CD21^low^ B Cells

A distinct B cell subset expressing low levels of the complement receptor 2 (CD21^low^B cells) is reportedly expanded in SSc patients compared to healthy controls (21). These cells are characterized by anergy and exhaustion, but they tend to be resistant to apoptosis when compared to healthy donors resulting in their expansion in the blood of SSc patients. Memory B cells represented the prominent B cell subset in patients with CD21^low^ B cells. DN CD27^−^IgD^−^ B cells were also increased in SSc -CD21^low^ patients compared to SSc-CD21^+^ patients. In contrast, naïve B cells represented the prominent B cell subset in SSc-CD21^+^ patients. CD21^+^ and CD21^low^ B cells from patients with SSc illustrated higher CD19 expression compared to similar subsets of healthy controls. DLCO was significantly decreased in SSc-CD21^low^ patients compared to SSc-CD21^+^ (p <0.01). Therefore, expansion of CD21^low^ B cells may define a distinct group of patients with SSc and vascular complications.

Among various clinical manifestations of SSc, only ILD was found to be associated with expansion of CD21^low^ B cells in the periphery (22). Such B cells in addition express lower cytoplasmic amounts of Bruton's tyrosine kinase (BTK) compared to normal B cells; in fact, their anergic state has been assigned to suppression of BTK-mediated signaling.

BTK is believed to be overexpressed in autoimmune diseases. A study examined the effects of ibrutinib, a BTK inhibitor, on B cells of SSc patients (23). High doses of ibrutinib (10 μM) resulted in decreased production of all the detected cytokines including IL-6, TNF-α, IFN-γ, and IL-10 and also in decreased production of anti-Scl-70 autoAb. Interestingly, low-doses of ibrutinib (1 μM) decreased the proinflammatory cytokines IL-6 and TNF-α only, increased the anti-fibrotic cytokine IFN-γ and finally it did not affect the anti-inflammatory cytokine IL-10. Ibrutinib treatment significantly decreased the levels of phosphorylation of the proinflammatory transcription factor NFκB that was previously induced with CpG stimulation. Furthermore, high doses of ibrutinib treatment also significantly reduced IL-6^+^ B cells among naïve and memory B cells, but the low doses of ibrutinib decreased the IL-6^+^ cells among naïve only, but not memory B cells, a finding of uncertain significance.

Another study disclosed that the CD21^lo/neg^ B cell subset that express low levels or lack surface CD21 was increased in SSc patients with ILD compared to those without ILD and healthy subjects, but frequencies of CD21^lo/neg^ B cells did not differ between patients with diffuse vs. those with limited disease (24). The DN B cell subset consisted of CD21^+^ and CD21^lo/neg^ B cells, but only CD21^lo/neg^ B cells were increased in patients with SSc-ILD. Histological examination of an explant from 1 patient with SSc-ILD and from 1 patient with chronic post-obstructive pneumonia disclosed that the patient with SSc-ILD had dense B cell infiltrates throughout all histological sections but the other patient had only 1 focus of B cells. Most B cells in the parenchyma of the lung did not stain for CD21, but the B cells in the germinal center did. Previous reports also suggest that CD21^lo/neg^ cells are localized into sites of inflammation. Further studies are necessary to define the role of CD21^low/neg^ B cells in the pathogenesis of SSc-ILD.

**Figure 1:**
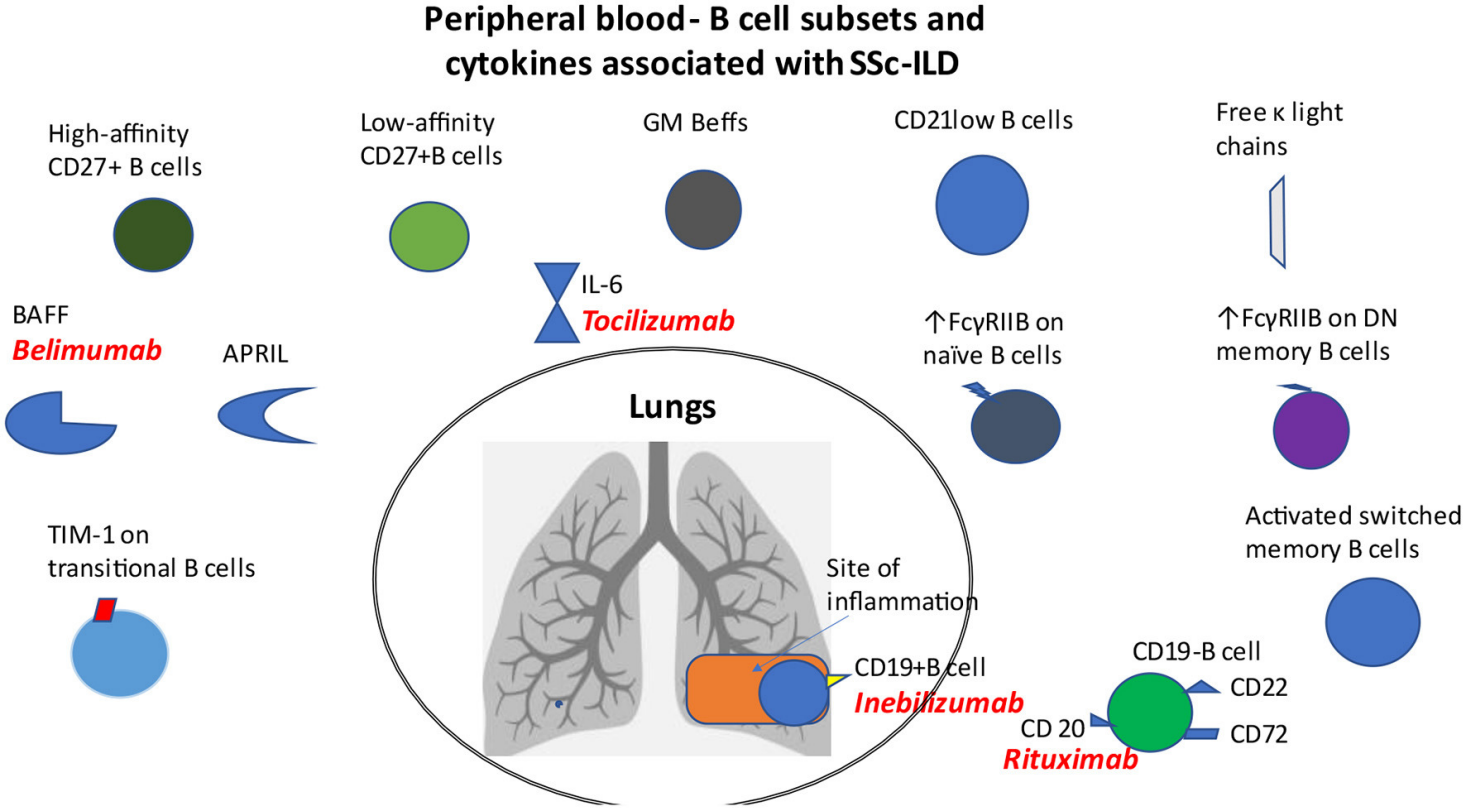
B cell subsets and cytokines in patients with SSc-ILD. Distinct B cell subsets such as CD21^lo^, GM-Beffs, activated switched memory B cells, topo I-reactive B cells with high affinity for topo I, increased expression of FcγRIIB on naïve B cells and that of TIM-1 on transitional B cells, as well as increased levels of free κ light chains, BAFF and APRIL cytokines are associated with ILD in patients with SSc. CD19 modulates the accumulation of B cells in BAL fluid. Loss of CD22 or CD72 decreased lung fibrosis.

Previously mentioned distinct B cell subsets that are associated with SSc-ILD are shown in Figure 1.

## D) Role of Cytokines Essential for B Cell Survival

### BAFF

Studies in TSK/+ mice have demonstrated elevated serum BAFF levels, although the expression of the 3 different BAFF-receptors, BAFF-R, TACI and BCMA was not altered (25). In an effort to block the effects of BAFF, 8-wk-old TSK/+ mice treated with BAFF-R-Ig displayed reduced hypodermal thickness by 49% compared to TSK/+ littermates, and also decreased cutaneous collagen content by 51%. Therefore, BAFF blockade via BAFF-R-Ig decreased fibrosis and skin collagen accumulation in TSK/+ mice. It was also shown that BAFF antagonism regulates B cell phenotypes in TSK/+ and wild-type littermates. More specifically, BAFF antagonism reduced total B cells by 75% in the spleen of TSK/+ and wild-type littermates and diminished mature B cells in the spleen and bone marrow; it also decreased transitional type 2 (T2 B cells), marginal zone and follicular B cells in TSK/+ and wild-type littermates. In contrast, BAFF antagonism significantly augmented transitional type 1 (T1 B cells). BAFF antagonism also decreased the levels of anti-topoisomerase I autoAb as well as the levels of IgM, IgG2a, IgG2b, and IgG3 in TSK/+ mice. In addition, it was reported that BAFF antagonism downregulates the previously amplified type 2 cytokines mRNA expression in TSK/+ mice, such as TGF-β, IL-6, and IL-10 and increased the expression of the anti-fibrotic cytokine IFN-γ in the skin of TSK/+ mice.

Median BAFF levels in the sera of patients with SSc were higher compared to healthy controls and significantly increased in patients with dcSSc compared to those with lcSSc (26). Of note, higher BAFF levels were observed also in patients with reduced FVC (*p* < 0.05), but the overall frequency of lung disease in dcSSc patients with increased BAFF levels was similar to those with normal BAFF levels. A 6-year follow-up study of 21 SSc patients illustrated stable BAFF levels in 52%, decreased BAFF levels in 33% and increased, over the time, BAFF levels in 14% compared to baseline levels. Among the 3 different receptors that bind BAFF, BAFF-R is the only one that specifically interacts with BAFF. BAFF-R expression on B cells of SSc patients was found to be increased compared to healthy controls. However, patients with SSc who were treated with belimumab, a mAb blocking soluble BAFF, did not exhibit improvements in FVC and DLCO during treatment (27).

A proliferation-inducing ligand (APRIL) is a cytokine demonstrating close homology with BAFF that has an important role in B cell development. Patients with SSc and SLE demonstrated increased serum APRIL concentrations compared to healthy controls (28). SSc patients with increased APRIL concentrations were more likely to develop pulmonary fibrosis than those with normal APRIL levels (65 vs. 37%, *p* < 0.05). Additionally, VC percentages in SSc patients with higher levels of APRIL were significantly reduced compared to those with normal APRIL levels (*p* < 0.05), despite the absence of differences in %DLCO among patients. Elevated levels of APRIL were specifically correlated with lung but not with other organ involvement. Increased APRIL levels were also correlated with hyper-γ-globulinemia, but not with SSc-specific autoAb levels or acute-phase protein levels. Furthermore, serum APRIL levels did not exhibit any association with BAFF levels. Separation of SSc patients in groups according to APRIL and BAFF concentrations illustrated that patients with high BAFF levels had a higher modified Rodnan skin score (*p* < 0.05), while patients with higher APRIL levels had significantly reduced %VC and %DLCO (*p* < 0.05).

## E) Evidence for the Role of B Cells in the Fibrotic Process

### B Cells and BAFF Promote Fibrosis

In one study dermal fibroblasts from SSc patients were cocultured with B cells from healthy controls (29). According to the results, B cells induced production of collagen by dermal fibroblasts of both healthy subjects and SSc patients with equal potency to that observed with the addition of TGF-β1 in cell cultures. Coculture of dermal fibroblasts with whole PBMC instead of B cells did not yield any differences in the production of collagen compared to culture of dermal fibroblasts without PBMCs. Coculture of B cells with dermal fibroblasts from SSc patients resulted in the induction of COL1A1, COL1A2, and COL3A1, whereas it increased levels of α-ASMA, TIMP1, and MMP9 expression. BAFF addition also induced the expression of collagen and of profibrotic markers. The use of transwells to prohibit cell-cell contact inhibited collagen production and profibrotic markers, while the addition of BAFF and anti-IgM to stimulate B cells, resulted in a partial inhibition by transwells. The addition of a neutralizing antibody against TGF-β1 inhibited collagen production effectively. Thus, direct cell-cell (B cell—dermal fibroblast) contact and profibrotic cytokines may be implicated in B cell- and BAFF- induced fibrosis, which is eventually mediated by TGF-β1.

### B Cells Secrete IL-6 and TGF-β and Activate Fibroblasts

Naïve, Breg, effector memory cell, and plasmablast counts were similar in the periphery of SSc patients and healthy controls (30). The proportion of IgD^−^CD27^+^ memory B cells was decreased in patients with SSc compared to healthy donors and the IgD^−^CD38^+^ early Bm5 subpopulation was the most prominently decreased subset. Early Bm5 memory cells express moderately positive levels of CD38 but they do not express IgD. B cells from SSc patients displayed increased expression of the activation markers CD95, CD69, and CD86 compared to healthy donors. A distinct B cell subset expressing both CD69 and CD95 in patients with SSc was not present in healthy controls. CD95^+^ B cell count was expanded in early limited SSC compared to those with not-early limited SSc and CD69^+^ B cell count was increased in early diffuse SSc compared to patients with not-early diffuse SSc. Additionally, the percentages of CD95^+^ and CD69^+^ B cells were increased in patients with early disease compared to those without early SSc. B cells that express CD22, CD32, and CD72 were similar between SSc patients and controls. Expression of CD20 on the surface of B cells was increased in patients with SSc compared to healthy controls. Similarly, there was a significant increase in the populations expressing IL-6R or IL-21R in patients with SSc compared to healthy donors but patients without pulmonary disease had a decreased proportion of B cells expressing IL-6R. Of note, the IL-6 receptor inhibitor, tocilizumab, an approved treatment for SSc-ILD, may stabilize lung function in early SSc-associated ILD over 48 weeks of treatment (31). A significant increase of IL-6-producing B cells was found in SSc patients compared to healthy controls. B cells from SSc patients had increased percentages of TGFβ^+^CD19^+^ B cells compared to healthy controls. B cells from SSc patients secreted increased concentrations of IL-6 and TGFβ and lower amounts of IL-10 upon stimulation, in contrast to a strong increase of IL-10 secretion that was seen in healthy controls after stimulation.

Increased levels of TGFβ were also measured in the supernatants of CpG-stimulated B cells from patients with SSc compared to healthy controls. Supernatants from CpG- or PMA-stimulated SSc B cells enhanced the proliferation of fibroblasts of healthy subjects and SSc patients. Increased expression of vimentin and mRNA for the transcription factor Snail and type I collagen was reported following co-culture of fibroblasts from SSc patients and healthy controls with CpG -stimulated B cell supernatants from patients with SSc. The transcription factor Snail and vimentin are proteins thought to be associated with the trans-differentiation of fibroblasts into myofibroblasts. In agreement with the above, the addition of anti-TGFβ or anti-IL6R mAb in cultures prohibited fibroblast proliferation.

## F) Autoreactivity of B Cells, Autoab Production and Free Light Chains

### Antigen Affinity in B Cell Autoreactivity

In systemic sclerosis specific autoAb are strongly correlated with distinct clinical phenotypes. Anti-topoisomerase I autoAb, also known as anti-Scl-70, are associated with diffuse cutaneous disease and pulmonary fibrosis. A study reported that frequencies of anti-topo I (+) CD27^+^CD19^+^ B cells were significantly expanded in anti-topo I autoAb-positive SSc patients compared to healthy controls and anti-CENP autoAb-positive SSc patients (32). Thus, topo-I-reactive CD27^+^ B cells are found only in anti-topo I autoAb-positive SSc patients. The majority of topo I-reactive CD27^+^ B cells produced one type of cytokines (IL-2, IL-6, IL-10, IL-14, IL-16, IL-23, IL-35, and TGF-β1). In contrast, topo I-non-reactive B cells did not express any of these cytokines. Therefore, one may hypothesize that topo I-reactive CD27^+^ B cells have already been stimulated by self-antigens *in vivo*. Association of the affinity magnitude of topo I reactive CD27^+^B cells with the cytokine production profile is provided in Table 2. The ratio of high-affinity CD27^+^ B cells to low-affinity CD27^+^ B cells correlated positively with the mRSS and inversely with the percentage predicted values of FVC and DLCO (*p* < 0.0001). Patients in the high-affinity-dominant group (with a frequency of more than 50% of B cells with high affinity for topo I) demonstrated longer disease duration, higher mRSS, frequent digital scars or ulcers and lower values of %FVC and %DLCO compared to low-affinity-dominant group (with a frequency of more than 50% with low affinity for topo I). Thus, topo I-reactive B cells with high affinity for topo I are associated with severe skin and lung fibrosis. BTK inhibition resulted in increased numbers of cells producing IL-10 and IL-35 and in amplification of IL-10 and IL-35 amount production, whereas the numbers of cells producing IL-6 and IL-23 were reduced. Therefore, BTK inhibition decreased the numbers and the affinity for topo I of topo I-reactive B cells, accompanied by an increased production of inhibitory cytokines, and finally to fibrosis attenuation. One study evaluated 10 patients with SSc treated with RTX; mRSS improved, but anti-topo I autoAb titers did not change. Therefore, one might conclude that it is cytokines and not autoAb production that is critical for the mechanism of BTK inhibitors in SSc pathogenesis. The presence of anti-topo I antibodies in SSc patients' sera could be considered as an epiphenomenon; however, it has been demonstrated that anti-topo I autoAb levels correlate with disease activity and severity. Thus, topo I-reactive B cells may participate in disease pathogenesis, regardless of the presence of anti-topo I autoAb.

**Table 2:** Association of the affinity magnitude of anti-topo I reactive CD27^+^ B cells with cytokine production profile.

**Topo I reactive CD27+ B cells**	**Cytokines**
Low affinity	IL-10
	IL-35
High affinity	IL-2
	IL-6
	IL-23
	TGF-β1

### Free Light Chains in SSc

Antibody production is accompanied by an increased synthesis of immunoglobulin light chains in the peripheral blood. Increased levels of free κ and λ light chains were found in the sera of SSc patients compared to healthy controls (33). Free κ serum levels were above the cut-off value of 19.4 mg/L in 57 out of 72 patients. The κ/λ ratio and κ+λ sum were reportedly increased in the sera of SSc patients compared to healthy donors. Additionally, free κ light chains were increased in the urine of SSc patients compared to healthy donors. Patients with increased κ+λ sum illustrated higher ESR, C-reactive protein, disease activity index, and disease severity scale scores compared to SSc patients with normal levels of free light chains. According to the results of the study, serum and urine free light chains produced by activated B cells may represent potential biomarkers of disease activity.

Another study examined the possible association of free light chains with organ involvement (34). Free κ light chains were increased in patients with disturbed lung function (FVC <80%): 26.4 ± 7.4 mg/L compared to patients with normal pulmonary function (FVC≥80%): 19.6 ± 7.3 mg/L. There was no difference in free light chains concentrations or κ/λ ratio between patients who had a deterioration of their pulmonary function and patients that had no deterioration. The association between increased levels of free light chains in SSc patients with pulmonary involvement might strongly suggest that B cell overactivation is implicated in disease pathogenesis.

## G) Indirect Evidence for the Roles Of B Cells

### B Cell Depletion Treatment in Models

The effects of B cell depletion have been evaluated in murine models of SSc. Low numbers of CD19^low^B220^+^B cells were found in the blood, spleen and lymph nodes following B cell depletion therapy (35). These cells were pre-B or immature B cells recently migrating from the bone marrow. B cell depletion treatment decreased dermal and hypodermal thickness in Tsk/+ mice by 31 and 43%, respectively, compared to control mAb treated animals, but it did not affect skin fibrosis in wild-type mice. B cell depletion treatment also decreased content of hydroxyproline by 50% in TSK/+ mice, although this remained significantly higher compared to wild-type littermates. B cell depletion may suppress skin fibrosis at the early stages of the disease process; however, in Tsk/+ mice, it does not affect established sclerosis or lung emphysema. Similarly, autoAb production and hypergammaglobulinemia were decreased when treatment was administered early but not later in the disease process. Tsk/+ and wild-type littermates were treated with anti-CD20 mAb or control mAb at days 3 and 17, with B-cell associated transcripts quantified in the skin from 3-wk-old- mice, at a time-point when blood and spleen CD19^+^B220^+^ B cells are eliminated by more than 93%. B cell transcripts were not detected in the skin of Tsk/+ as well as in wild-type littermates. B cell depletion treatment also regulated the cytokine transcript profile in the skin and spleen of the experimental animals Table 3.

**Table 3:** Effects of B cell depletion treatment on the cytokine profile measured at the skin and spleen of TSK/+ mice.

Skin	TGF-β transcripts	↓
	TNF-α, IL-2, IFN-γ mRNA	↑
	IL-4, IL-6 IL-10 mRNA	↓
Spleen	IL-2, IL-4, IFN-γ mRNA	↑
	IL-6, IL-10, TNF-α mRNA	↑
	TGF-β mRNA	–

*↑, Increased levels; ↓, decreased levels; –, stable*.

### BAFF Inhibition

A study suggests that BAFF inhibition diminishes fibrosis by affecting the balance between effector and regulatory B cells (Beffs and Bregs, respectively). IL-6- and IL-10-producing Beff counts were found to be increased in inflamed skin of mice treated with bleomycin compared to those treated with phosphate-buffered saline (36). IL-6 deficient B cells induced an attenuation of dermal thickness, collagen expression and lung fibrosis. It is clearly important that IL-6-producing Beffs mediate fibroblast collagen production.

BAFF induced IL-6 production from B cells after stimulation with LPS and/or CD40 and at the same time inhibited IL-10 production from B cells following stimulation with LPS. In addition, BAFF resulted in expansion of IL-6-producing Beffs but at the same time in elimination of IL-10-producing Bregs. Importantly, not only skin but also lung fibrosis of mice treated with the BAFF-blocker BAFFR (BAFF-Receptor)-Fc was significantly diminished compared to those treated with Fc control protein. BAFFR-Fc led to reduced IL-6- producing Beff counts but didn't alter numbers of IL-10-producing Bregs. IL-6-producing Beffs are considered pathogenic, but IL-10-producing Bregs are proposed to have a protective role.

### Role of Anti-CD22 AutoAb

A study assessed the effect of autoAb against CD22 present in the sera of SSc patients as well as the sera of TSK/+ mice (37). Titres of anti-CD22 autoAb were higher in the sera of patients with SSc compared to healthy controls (where they were undetectable), and they were higher in patients with diffuse compared to those with limited SSc. Of note, titres of anti-CD22 autoAb were higher in patients with SSc compared to patients with SLE.

Surfactant protein D (SP-D) plays an important role in lung homeostasis due to its immunomodulatory and antiviral properties. It was demonstrated that patients with anti-CD22 autoAb exhibited higher SP-D concentrations compared to those without anti-CD22 autoAb [SP-D: 137 (44–502) ng/dl vs. 73.2 (29.2–311) ng/dl, *p* < 0.05]. Of note, anti-CD22 autoAb were correlated with lower values of vital capacity percentage (%VC) compared to anti-CD22 (–) negative patients, but the difference was not statistically significant (78.2% vs 99.2%). Furthermore, anti-CD22 autoAb reduced CD22 tyrosine phosphorylation by Lyn kinase upon BCR ligation, but this finding was not specific for patients with SSc. Additionally, in the presence of anti-CD22 autoAb the tyrosine phosphorylation of CD19 was augmented following BCR ligation. Thus, one might hypothesize that B cells produce anti-CD22 autoAb which in turn activate B cells in an autocrine manner leading to the perpetuation of the immune response.

### Autologous Hematopoietic Stem Cell Transplantation Effects on B Cells

Alterations in B cell subsets that sustained up to 14 months following autologous hematopoietic stem cell transplantation (aHSCT) were reported in 6 SSc patients (38). Regarding lung involvement data were evaluated before and at 10–18 months after aHSCT. The median percentages of DLCO and FVC remained stable during the study (*p* = 0.315 and *p* = 0.066, respectively). On the functional side, another study suggests that aHSCT may restore the previously reduced inhibitory function of Bregs in SSc patients (39). The effects of aHSCT on B cell subsets and cytokine production in patients with SSc are summarized in Table 4.

**Table 4:** Autologous hematopoieitic stem cell transplantation effects on B cell subsets and cytokine production in patients with SSc.

CD38^++^/CD10^+^/IgD^+^ transitional	↑
CD38^++^/CD27^++^/IgD^−^ plasmablasts	↑
CD27/IgD^+^ naïve	↑
CD27^+^/IgD^+^ pre-switched memory	↓
CD27^+^/IgD^−^ post-switched memory	↓
CD27^−^/IgD^−^ DN	↓
Plasma cell	↓
IL-10 producing Breg cells^*^	↑
IL-6 and TGF-β producing B cells	↓
Th1 cytokines^**^	↓
IL-10	↑

*↑, Increased; ↓, Decreased*.

* *Defined as: CD19^+^CD24^hi^CD38^hi^ and CD19^+^CD24^hi^CD27^+^ cells*.

** *Because CD19^+^CD24^hi^CD38^hi^ Bregs recovered their ability to inhibit Th1 cytokine production by CD4^+^ T cells*.

## Discussion

A plethora of B cell abnormalities render B cells important players of SSc pathogenesis. The expression of activating and inhibitory molecules tuning B cell functions in B cell subsets has reportedly been disturbed. Studies in murine models of SSc illustrated that CD19 signaling pathway in B cells plays a critical role in the development of skin fibrosis. B cells in Tsk/+ mice upregulated CD23 expression and downregulated surface IgM expression, amplified constitutive CD19 and Vav tyrosine phosphorylation, enhanced the activity of kinase Lyn and also augmented CD19-induced [Ca^2+^] responses, hyper-γ-globulinemia and response to BCR ligation. Importantly, CD19 deficiency resulted in the reduction of collagen deposition; yet, CD19 overexpression did not amplify skin fibrosis in Tsk/+ mice. It was also proposed that CD19 levels modulate the accumulation of B cells in BAL fluid suggesting thereby a role of CD19 signaling in the development of lung fibrosis.

The inhibitory regulation of CD22 was found to be disturbed in B cells of Tsk/+ mice. The CD19/CD22 signaling loop is strongly correlated with anti-topo I autoAb production. Loss of inhibitory molecules, either CD22 or CD72, decreased skin and lung fibrosis revealing a role for both negative co-receptors of BCR signaling in disease pathogenesis. It has been also proposed that B cells produce anti-CD22 autoAb leading to a vicious circle of B cell activation. Anti-topo I autoAb may not have a direct pathogenic role in the development of fibrosis in SSc. Studies in humans revealed that increased peripheral B cells in patients with SSc exhibit increased expression of activation markers, cytokine receptors and increased levels of IL-6 and TGF-β. Mice with a B cell-specific deficiency in IL-6 exhibited attenuation of their skin and lung fibrosis, whereas those with a B cell-specific deficiency in IL-10 had severe skin and lung fibrosis. BAFF antagonism decreased skin and lung fibrosis in the bleomycin-induced scleroderma model via reduction of Beffs. B cells may increase the expression of FcγRIIB in order to counterbalance their abnormal hyperactivation. Moreover, FcγRIIB expression levels may represent a marker of disease severity, mainly of ILD.

B cell homeostasis also seems to be impaired. CD21^lo^ B cells have been found increased in SSc patients with lung involvement. High counts of GM-Beffs were found to be associated with the diffuse form of SSc with concomitant ILD. The association of activated switched memory B cells with the presence of anti-topoisomerase I autoAb may reflect their potential role in the abnormal autoAb production. Reduced Breg cells were associated with lung disease. Free light chains production by immunoglobulins that reflect B cell overactivation have been found in high concentrations in the sera of patients with SSc and are associated with lung involvement. Disrupted removal of autoreactive naïve B cells may result in the production of self-antigen specific B cells that produce autoAb. Increased BAFF levels were correlated with disease severity, whereas decreased BAFF levels were correlated with attenuation of skin fibrosis. B cells that had been stimulated with BAFF were found to have an amplified capacity to secrete IL-6 and IgG. Profibrotic cytokines and direct cell-cell contact may be participate in TGF-β1-mediated-B cell- and BAFF-induced fibrosis. Increased levels of APRIL in the SSc patients' sera were associated with a greater incidence of lung fibrosis.

Indirect evidence for the role of B cell in SSc pathogenesis result from the efficacy of the anti-CD20 mAb in patients with SSc. A 1-year proof-of-principle study showed that patients treated with rituximab experienced an improvement of lung function (mean FVC ± S.D.: 68.13 ± 19.69 vs. 75.63 ± 19.73, at baseline vs. 1 year, *p* = 0.0018 and mean DLCO ± S.D.: 52.25 ± 20.71 vs. 62 ± 23.21, at baseline vs. 1 year, *p* = 0.023) (40). In contrast, patients that were not treated with rituximab had a deterioration of their pulmonary function. Platelet-derived growth factor (PDGF) participates also in fibrotic process in SSc. A study demonstrated that PDGFRα and PDGFRβ expression as well as phosphorylated PDGFα and PDGXβ expression in the papillary dermis were significantly reduced following rituximab treatment (41). Therefore, rituximab may improve skin fibrosis by altering the pathway mediated by PDGF via unknown mechanisms. Autologous hematopoietic stem cell transplantation causes alterations in B cell subpopulations and seems to restore the previously decreased suppressed function of Breg cells in patients with SSc.

The question on how autoimmunity leads to the clinical phenotypes of SSc, in particular to lung disease, cannot be answered yet. Recently, some light has been shed on B cell abnormalities that are present in patients with SSc and participate in disease pathogenesis through poorly understood mechanisms. However, B cell targeting therapies could be integrated into the therapeutic armamentarium of patients with resistant SSc-ILD aiming to at least stabilize the fibrotic lung process.

## Author Contributions

S-NL conceived and wrote and edited the manuscript. CS wrote and edited the manuscript. All authors contributed to the article and approved the submitted version.

## Funding

The publication of this article has been financed by the Research Committee of the University of Patras.

## Conflict of Interest

The authors declare that the research was conducted in the absence of any commercial or financial relationships that could be construed as a potential conflict of interest.

## Publisher's Note

All claims expressed in this article are solely those of the authors and do not necessarily represent those of their affiliated organizations, or those of the publisher, the editors and the reviewers. Any product that may be evaluated in this article, or claim that may be made by its manufacturer, is not guaranteed or endorsed by the publisher.
